# Pharmacoepidemiological assessment of adherence and influencing co-factors among primary open-angle glaucoma patients—An observational cohort study

**DOI:** 10.1371/journal.pone.0191185

**Published:** 2018-01-12

**Authors:** Stefanie Frech, Daniel Kreft, Rudolf F. Guthoff, Gabriele Doblhammer

**Affiliations:** 1 Department of Ophthalmology, Rostock University Medical Center, Rostock, Germany; 2 Institute for Sociology and Demography, University of Rostock, Rostock, Germany; 3 Rostock Center for the Study of Demographic Change, Rostock, Germany; 4 German Center for Neurodegenerative Diseases (DZNE), Bonn, Germany; Bascom Palmer Eye Institute, UNITED STATES

## Abstract

The goal of this study was to assess the adherence of primary open-angle glaucoma (POAG) patients to medication, and to determine co-factors influencing adherence, using a representative sample of members of the largest German public health insurer. The observational cohort study was based on a longitudinal data set from 2010–2013 and included 250,000 insured persons aged 50 and older with 10,120 diagnosed POAG patients. Uni- and multivariate analysis was performed to investigate several aspects of glaucoma, such as prevalence, adherence, and co-factors influencing adherence. The main outcome measured adherence with prescriptions filled within a year. Multivariate panel regression analysis was used to determine the co-factors influencing this adherence. Prevalence of POAG was 3.36% [CI: 3.28–3.43%], with 2.91% [CI: 2.81–3.01%] for males and 3.71% [CI: 3.61–3.81%] for females, increasing with age. The mean level of adherence in terms of prescriptions filled was 66.5% [CI: 65.50–67.60%]. The results of this analysis revealed a significant influence of age, duration of the disease, care need, distance to death, and multimorbidity as co-factors of non-adherence, whereas gender had no influence. The analysis provided detailed information about POAG health care aspects concerning prevalence and adherence. The most endangered risk groups for non-adherence were patients aged 50–59, patients older than 80 years, patients with a longer duration of POAG, patients with care needs, and patients with three or more severe diseases in addition to glaucoma. To know the predictors responsible for an increased risk to develop POAG is of importance for all persons involved in health care management. Therefore effective strategies to increase awareness of patients and medical care personnel about non-adherence and the importance of a regular and continuous medication to avoid further nerve fiber damage and possible blindness have to be developed.

## Introduction

Primary open-angle glaucoma (POAG) is a chronic and initially asymptomatic disease causing early irreversible nerve fiber damage. The disease progresses slowly without any notable sign of vision loss until it is very advanced. If an appropriate treatment strategy is initiated early enough, glaucoma blindness can be prevented in most patients [1, 2].

Worldwide, glaucoma is the second leading cause of blindness and primary open-angle glaucoma (POAG) is expected to affect 58.6 million people by 2020. The global mean prevalence of POAG in 2010 was 1.96%, with more women affected than men. In Europe, glaucoma prevalence is higher than 5% for patients aged 80 and older [3] but reaches much higher levels in other regions and populations of the world. For example, an urban West African population study, the Tema Eye Survey, found POAG prevalence values of 6.8% for those aged 40 and older, increasing to 14.6% among those 80 years and older [4]. In the USA, the Salisbury Eye Evaluation Glaucoma Study determined prevalences of 3.4% for white individuals aged 73 and 74, and of 9.4% for people aged 75 years and older. Among black individuals the prevalence was 5.7% for those aged 73 and 74, increasing to 23.2% for people aged 75 and older [5].

Population ageing reinforces the importance of early and adequate treatment of POAG in order to prevent visual impairment and decline in quality of life. However, treatment is often hampered by the fact that POAG is an asymptomatic disease where an immediate benefit and improvement of symptoms is not obvious.

There are two major strategies for lowering and permanently controlling intraocular pressure (IOP): surgical interventions or topical local medication. It is known that using drops once or even several times a day poses a major challenge for many patients [6]. This leads to the risk of patients not being adherent, which in turn reduces treatment effectiveness. Adherence is a measure of the degree to which a patient follows the prescribed instructions during a defined time period [7]. Improving adherence to the set therapeutic regimen is one of the major challenges for glaucoma management. Patients being treated either by laser and more invasive surgical interventions have been excluded for the evaluation of adherence.

Reasons for non-adherence are multifactorial and include knowledge about and difficulties with self-administration, forgetfulness, disturbing side effects, and levels of education [8–12]. Tsai et al. (2003) identified and systematically classified a number of adherence barriers, defining four major categories: environment-related, patient-related, treatment-related, and provider-related [13]. Of these four adherence barriers, it is the category of patient-related factor which is of interest in this study.

It is not easy to obtain accurate and precise measurements of adherence, and previous studies have employed various techniques to investigate and describe adherence. Assessment techniques include questionnaires [8, 12] and interviews [13–15], patient chart reviews [16], medication monitors [17, 18], and investigations of administrative [16, 19] and insurance claims databases [19–22]. A systematic review by Lu et al. revealed that non-adherence was an issue in more than 25% of all POAG patients [23], while others describe even higher proportions of non-adherence, up to 50% [20], between 25–50% [9], and as high as 51% [19].

Eye disorders are among the top five conditions in which progression is closely related to poor patient adherence. The proportions are comparable to those with oral medication for hypertension and other chronic asymptomatic conditions [24]. It has been estimated that only 50% of patients with chronic illnesses in developed countries adhere to their medication, and the problem is expected to be much more pronounced in developing countries [25].

This study used a database of a large German health insurance provider to investigate the non-adherence of POAG patients. The objective of analyzing these data was to evaluate the collection proportions of topical eye drops from the pharmacy by analyzing the number of prescriptions which were actually filled. In order to identify the most endangered subgroups of non-adherent patients, the influence of potential patient-related co-factors was investigated using a multivariate panel regression model.

## Material and methods

### Data source

A random sample of 250,000 members born in 1960 and earlier who were living in private households and institutions was taken from the database of the largest German public health insurance agency, the Allgemeine Ortskrankenkasse (AOK). The observation period ranged from the first quarter of 2010 to the last quarter of 2013, or until an earlier exit from the study due either to a change in health insurance or to death. The quarterly individual-level data covered general demographic data, inpatient and outpatient diagnosis data coded by the International Classification of Disease 10th revision (ICD-10), medical treatment data coded by the German Procedure Classification (OPS), and prescriptions of medications coded by the German Anatomical Therapeutic Chemical (ATC)-Classification. Antiglaucoma medication (AGM) was defined by the ATC codes S01EA-E and S01EX.

Access to the data was granted and approved by the Scientific Institute of the AOK (WIdO). All analyses of this study are based on anonymized administrative claims data that never involved patients directly. Individual persons cannot be identified, and the results presented here do not in any way affect persons whose anonymized records were used. No ethical approval was required, therefore this study complies with the tenets of the Declaration of Helsinki.

### Study sample and validation process

**Fig 1:**
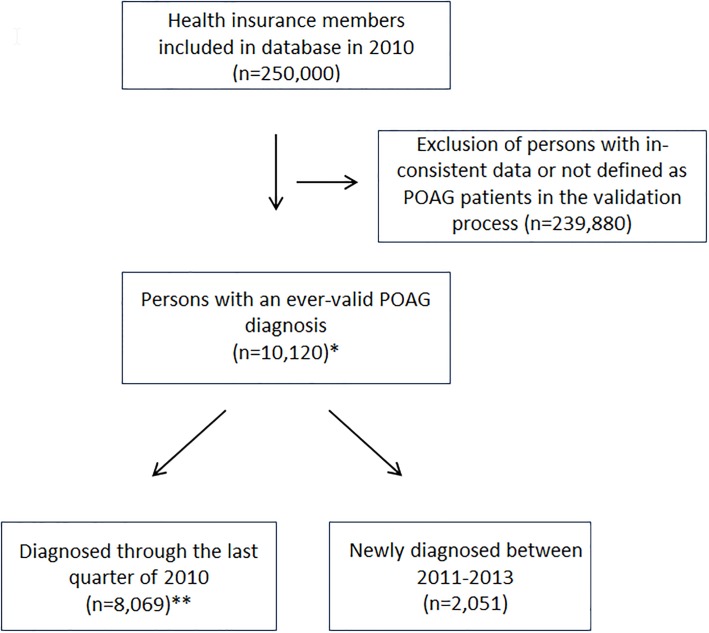
From survey sample to study sample—Flowchart of the selection procedure. * Basis for the multivariate analysis. ** Basis for the estimation of point prevalence and adherence in the last quarter of 2010. The group consists of persons ever valid diagnosed with POAG from birth until the last quarter of 2010 (prevalence).

For the analysis, all data were extracted from the database. All persons for whom information about year of birth and death was implausible or inconsistent were excluded from the analysis. POAG diagnosis was defined by ophthalmologists according to the ICD-10 code H-40.1, and validation required at least two POAG diagnoses within the observation period. The analysis sample comprised 10,120 persons who had ever experienced a validated POAG diagnosis, of which 8,069 persons had at least one valid diagnosis in 2010 and 2,051 persons had a valid POAG diagnosis in the years 2011 to 2013 (Fig 1).

To avoid end-of-study bias, the last two quarters of 2013 were excluded due to the short period for newly diagnosed POAG patients to receive a second confirmatory diagnosis. POAG was assigned from the first diagnosis onwards. The H40.1 code exclusively labels glaucoma patients for whom treatment is required. Suspected glaucoma and ocular hypertension patients were excluded from this study.

### Control variables

Our study controlled for gender and age groups (50–59, 60–69, 70–79, 80+) at entry into study, time in the study since the first validated POAG diagnosis, care level, multimorbidity, and the vital status.

Time in the study was measured in years (first, second, and third year after the first valid diagnosis). Because we excluded the last two quarters of 2013, the fourth year of observation was incomplete (“third year+”).

Care level was the grade of disability stated as evaluated by medical experts from the medical advisory services of the German association of statutory health insurance funds. Care level is divided into three categories, according to the severity of disability.

Multimorbidity status measured the number of diagnoses of the following selected severe diseases ever received: diabetes mellitus, acute myocardial infarction, cerebrovascular diseases, ischemic and other heart diseases, cancer, kidney diseases, lung diseases, dementia, and injuries of the lower extremities, hips and pelvis. Each diagnosis was validated—similar to the validation strategy for glaucoma diagnosis—by the occurrence of at least two diagnoses within the full observation period. All variables were combined into a score variable and categorized into four categories: None of the selected severe diseases, one disease, two diseases, and three or more diseases. The vital status is an indicator for the last year that a person was alive.

### Prevalence and adherence definitions

Age- and gender-specific point prevalences of POAG were estimated for the last quarter of 2010. Prevalence was defined as the age- (a) and gender-specific (x) proportion of persons who had a POAG diagnosis at any time in 2010 (Pre), who survived at least to the last quarter (Q4) of 2010, and who had a second diagnosis in at least one of the following quarters through the end of 2013 of the study population (Pop) with the same gender and age (Eq 1).

$${\text{Point}\;\text{prevalence}}_{\text{Q}4,2010,\text{x},\text{a}}=\frac{{\text{Pre}}_{\text{Q}4,2010,\text{x},\text{a}}}{{\text{Pop}}_{\text{Q}4,2010,\text{x},\text{a}}}∙100$$ (1)

Adherence was indicated on the basis of prescriptions filled. According to the German health care system, every diagnosed glaucoma patient is advised to be examined on a quarterly basis by an ophthalmologist. These visits include eye examinations and a new prescription for topical medication for the next three months. The quarterly information about the prescriptions which were filled was used as a basis for the calculation of adherence. A patient is free to visit the ophthalmologist several times within 3 month, but receives just one prescription per quarter. Changes in the specific type of AGM have no effect on the calculation of adherence, because all AGM were considered as one single group without sub-stratification.

Adherence was defined on a yearly basis. A patient was considered to be adherent for a full year the medication was picked up in a minimum of three of the four quarters of the year. An additional time frame of three quarters was included into this definition, as the quarterly amount of eye drops might last longer than one particular quarter. By the same definition, a patient was non-adherent if the prescription was picked up in only two or fewer of the four quarters in a one-year period. The level of adherence was estimated for the last quarter (Q4) of 2010 by dividing the number of adherent POAG patients (Adh) by age (a) and gender (x) by all patients diagnosed with POAG and the same gender and at the same age (Eq 2).

$${\text{Adherence}}_{\text{Q}4,2010,\text{x},\text{a}}=\frac{{\text{Adh}}_{\text{Q}4,2010,\text{x},\text{a}}}{{\text{Pre}}_{\text{Q}4,2010,\text{x},\text{a}}}∙100$$ (2)

In a sensitivity analysis, the influence of a different definition of adherence on the level of adherence and on the results of the multivariate analysis was tested. Adherence was alternatively defined on a quarter-specific basis according to the patient having filled a prescription in a specific quarter or not.

### Multivariate analysis

The risk of being adherent was modelled by applying random effects panel regressions with logistic link functions and a first-order autoregressive error term structure. This type of model is used for analyses based on panel data and aims to estimate the simultaneous effects of multiple time-varying and time-constant factors on a time-varying output variable. Since the output variable is dichotomous, a binomial distribution is expected and a logistic link function is specified. On the individual level, the quarterly observations are highly correlated. To face the problem of autocorrelation, a first- order autoregressive error term structure is specified that adjusts for the dependencies of observed attributed of individual persons at one quarter with observed attributes in the prior quarter. These specifications allows to estimate coefficients that can be generalized beyond the sample used in the analyses and to produce correct confidence intervals that take the panel structure with dependent observations into account [26].

The quarterly outcome variable was adherence yes/no, defined as one and zero respectively. Only persons with a validated diagnosis of glaucoma were tracked from the first diagnosis onwards, and these 10,120 persons were observed for an average of 11.7 quarters. While gender and age at entry were time-constant variables, all other variables were allowed to vary over time. According to the usual practice in epidemiological and health science, the logistic regression results were reported as odds ratios (OR). All estimations were made using the xtlogit routine by Stata version 12.1.

## Results

### Point prevalence of POAG

**Fig 2:**
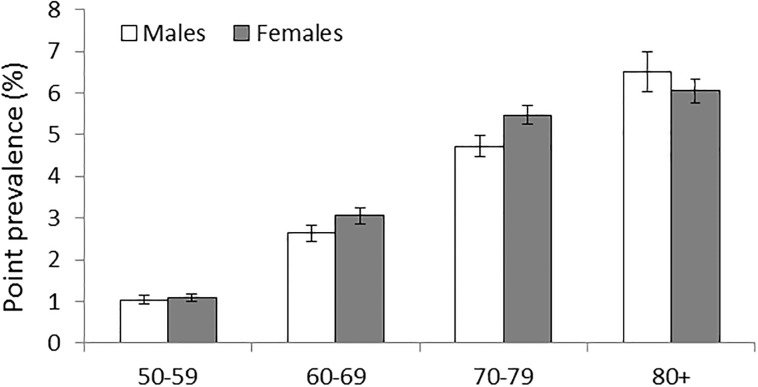
Prevalence of POAG by age groups and gender in 2010 including 95% CI, AOK data.

Prevalence of POAG in the last quarter of 2010 was 3.36% [CI: 3.28–3.43%] for the total study cohort. It was 2.91% [CI: 2.81–3.01%] for males and 3.71% [CI: 3.61–3.81%] for females. This prevalence increased with increasing age. At age 50–59 it was 1.04% [CI: 0.94–1.14%] for males and 1.09% [CI: 0.99–1.19%] for females; at age 80+ it reached a peak of 6.51% [CI: 6.04–6.99%] for males and 6.05% [CI: 5.77–6.34%] for females (Fig 2).

### Adherence indicated as proportion of filled prescriptions

**Fig 3:**
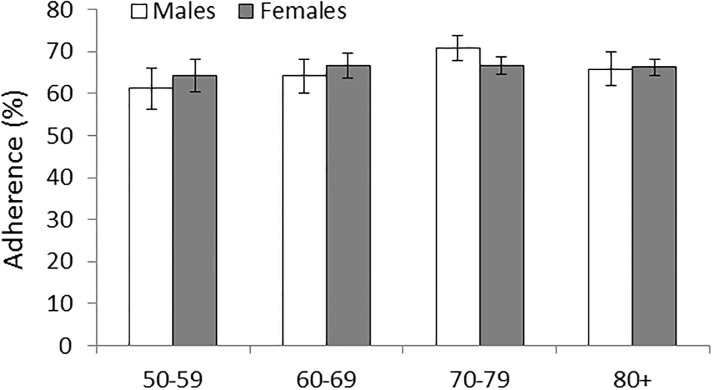
Percentage distribution of adherence by age groups and gender including 95% CI, AOK data.

Adherence was assessed by the proportion of prescriptions which were filled, adjusted for gender and age group (Fig 3). Total adherence was 66.5% (CI: 65.5–67.6%), with no disparity between genders (66.8% [CI: 65.2–68.5%] for males versus 66.3% [CI: 65.0–67.6%] for females). No age effect was detected for females, but for males a significantly higher adherence was measured at ages 70–79 (70.8%, CI: 68.6–73.3%) compared to ages 50–59 (61.2%, CI: 56.7–65.8%) and 60–69 (64.2%, CI: 60.6–67.7%).

### Characteristics of study population

For descriptive purposes all characteristics, with the exception of the quarter of death and the time since entry into the study, are measured at the first validated diagnosis. Patients aged 70–79 represent the largest group (40.7%) and those aged 50–59 the smallest (12.4%); 62.0% were female. The vast majority of patients (89.7%) had no care level at all, 7.0% had care level 1, 2.9% had care level 2, and 0.4% were assigned to the highest care level 3. The multimorbidity status revealed that 25.6% of the patients had none of the severe diseases listed above, 27.7% had one, 20.2% had two, and 26.5% had at least three severe diseases. Of the 10,120 patients, 981 (9.7%) died during the observation period. The total number of patients in the study decreased by 20%, from 10,120 to 8,278 people. This was primarily due to deaths, but some patients changed their insurance providers in this time period (Table 1).

**Table 1:** Descriptive overview of POAG patient characteristics during the observation period 2010–2013.

			Persons	%
At time of entry into study	Age group	50–59	1.254	12.4%
		60–69	2.137	21.2%
		70–79	4.115	40.7%
		80+	2.614	25.8%
	Gender	Male	3.849	38.0%
		Female	6.271	62.0%
	Care level	No	9.076	89.7%
		Care level 1	704	7.0%
		Care level 2	295	2.9%
		Care level 3/3+	45	0.4%
	Multimorbidity status	No	2.587	25.6%
		1 severe disease	2.804	27.7%
		2 severe diseases	2.049	20.2%
		3+ severe diseases	2.680	26.5%
Over the time of study	Vital status	Alive	9.139	90.3%
		Dead	981	9.70%
	Year of observation	1^st^ year*	10.120	
		2^nd^ year*	9.446	
		3^rd^ year+*	8.278	
	Total		10.120	100.0%

*1^st^ quarter of the year of observation

### Multivariate analysis of co-factors influencing adherence

Factors influencing adherence are shown in Table 2. Adherence increased up to the age of 70–79 where it was 20.9% higher (OR = 1.21, p-value<0.01) than for those aged 50–59. At ages 80+, adherence then decreased again to the level of those aged 50–59 (OR = 1.08, p-value = 0.22).

**Table 2:** Summary of the panel regression model with identified co-factors of non-adherence on a yearly basis, AOK data.

Characteristics		Odds ratio	p-value	Confidence interval
Age at time of entry	50–59	1.00		
	60–69	1.13	0.05	(1.00–1.26)
	70–79	1.21	0.00	(1.09–1.35)
	80+	1.08	0.22	(0.96–1.21)
Year after 1^st^ diagnosis	1^st^ year	1.00		
	2^nd^ year	0.80	<0.01	(0.79–0.81)
	3^rd^ year+	0.72	<0.01	(0.70–0.73)
Gender	Male	1.00		
	Female	0.98	0.50	(0.91–1.05)
Care level	Not disabled	1.00		
	Care level 1	0.90	<0.01	(0.86–0.94)
	Care level 2	0.79	<0.01	(0.74–0.85)
	Care level 3/3+	0.82	0.00	(0.73–0.92)
Multimorbidity status	No	1.00		
	1 severe disease	1.00	0.96	(0.95–1.05)
	2 severe diseases	0.97	0.25	(0.92–1.02)
	3+ severe diseases	0.94	0.03	(0.89–0.99)
Vital status	Alive	1.00		
	Year of death	0.72	<0.01	(0.68–0.76)

Time after the first diagnosis, which was associated with duration of the disease, was negatively related to adherence. Adherence was 20.0% lower (OR = 0.80, p-value<0.01) in the second year compared to the first year; it was 28.5% lower (OR = 0.72, p-value<0.01) in the third and in the following year compared to the first year.

There was no significant difference between genders, but an important factor of adherence was care need. Compared to persons without any need of care, those with care level 1 had 10.2% lower adherence (OR = 0.90, p-value<0.01), persons with care level 2 had 20.7% lower adherence (OR = 0.79, p-value<0.01), and persons with care level 3+ an 18.2% lower (OR = 0.82, p-value<0.01) adherence.

Multimorbidity contributed significantly to lower adherence. Those patients who had three or more severe diseases in addition to POAG had 6.1% lower (OR = 0.94, p-value = 0.03) adherence compared to persons without any of these diseases. In the year of death, adherence was 28.4% lower (OR = 0.72, p-value<0.01) than in the previous years.

### Results of the sensitivity analysis

In the sensitivity analysis, where adherence was defined on a quarter-specific basis, adherence values totaled 65.2% (CI: 64.2–66.3%), 66.0% (CI: 64.3–67.6%) in males and 64.8% (CI: 63.5–66.1%) in females.

The panel regression resulted in similar odds ratios of adherence (see Supplement material, S1 Table), with the exception of a small albeit significantly lower adherence in females (OR = 0.94, p<0.01) compared to males. In the last quarter in which patients were alive, adherence was 70.3% lower (OR = 0.30, p<0.01) than in previous quarters.

## Discussion

In this study, data from a German health insurance company were analyzed to estimate prevalence and investigate adherence factors of glaucoma patients. The number of prescriptions filled within a year was used to specify the adherence. The total level of adherence was 66.5%. By performing a multivariate panel regression analysis, the factors influencing adherence were determined to be age, duration of disease, care need, and multimorbidity. For Germany, data on the epidemiology of glaucoma and glaucoma management are rare. The data are extracted from a database with a large sample size which is representative of a German population.

Adherence is a multifaceted issue [27], dependent on different aspects of medication, starting with the appointment with a doctor and proceeding to prescription pickup and taking regular medication. Nevertheless, when comparing different adherence studies, it is important to consider that the methods and the observed time frames differ, which might result in different values of adherence [28].

To determine aspects of adherence many studies rely on insurance claims databases, which provide large samples of information and are generally considered valid in indicating proportions of adherence with glaucoma drugs [7]. Gurwitz et al. performed a study of 2,440 patients older than 65 years, measuring non-adherence by checking prescriptions filled for glaucoma medication over a period of 12 months. They found that 23% of patients were non-adherent, compared to 33.5% of non-adherent patients in our study. In both studies, gender was not found to be a predictor of non-adherence,. Nordstrom et al. used health insurance claims data to calculate the prevalence of medication adherence, with the result that nearly half of those patients who had filled a prescription discontinued the therapy within six months [20]. Duration of the disease was a predictive factor of the model analysis performed in this study. Similar to Nordstrom et al. a significant non-adherence was found starting after 12 month of medication. In a retrospective study Wilensky and colleagues extracted data from a pharmacy claims database to assess adherence of prescribed prostaglandin medication, calculating a mean proportion of 76% [29], which was similar to the mean adherence of 66.5% found in this study.

In this respect, it is important to consider ethnical and cultural differences. Rees and colleagues determined adherence proportions of glaucoma patients from diverse cultures (white Americans, African Americans, white Australians, Singaporeans of Chinese decent) and found significant differences in self-reported adherence proportions, varying from 65.4% to 47.5% [30]. Thus it seems that critical predictors of adherence differ, depending on living conditions and the cultural environment. This is why a direct comparison of data is difficult, as our results reflect a European health care management system, which is different than systems such as those in the USA or the United Kingdom, in which individual costs are a major part of non-adherence in addition to the aspects listed above [31, 32, 33]. In Germany, the majority of inhabitants (90%) are members of a statutory health insurance. In this study only those had been included. The medication cost is covered by the insurer and patients have only a small amount of co-payment on filled prescriptions (approx. 5€). Despite ethnological and health insurance differences, the percentage of non-adherent patients is astonishing similar. Even the German system, where the patients do not have to carry any extra financial burden, is not able to increase patient adherence. Whether there is a ubiquitous lack of personal responsibility towards one’s own health is a matter of discussion, but on presently available data still speculative.

As shown, the risk of non-adherence increased with the duration of the medication. In the second year the risk of non-adherence increased by 20%, in the third year it increased further to 28.5%. In general, monitoring adherence to medication is essential for the effective treatment of chronic asymptomatic diseases, such as glaucoma, diabetes, or hypertension. Because there are few or no symptoms, patients often do not realize the importance of taking daily medication [34]. According to Brown et al., evidence from a number of studies suggests that as many as 50% or even 80% of patients treated for hypertension are non-adherent to their treatment regimen. The adherence of patients who have been prescribed statin medication decreases between 6 months to 1 year, as 25% to 50% of patients discontinue them [35]. In a study of 500 drug-eluting-stent recipients, 13.6% of patients discontinue thienopyridine therapy within 30 days. The mortality rate for these patients was 10-fold greater at 1 year than for those who did continue the therapy [35]. Adherence with cardiovascular medication in terms of prescriptions filled was 72% in the first year of treatment in a one-year study [36]. These adherence numbers are very similar to the results found in this study which implies an overall level of non-adherence across different chronic diseases.

Compared to oral medication, adherence to locally ophthalmic medication poses some challenges, including manual dexterity, hand-eye coordination, and sufficient visual acuity, all of which tend to decrease with age [34]. Prevalences, as found in this study, increase steadily until the age of 80. Therefore, approaches need to be found to support older patients who face these hurdles. Achieving satisfactory adherence may be a more powerful tool than any other intervention to improve the therapeutic outcome [27].

In our study, a correlation for a higher risk of non-adherence with increasing numbers of severe diseases in addition to glaucoma was found. Other studies demonstrated that patients with multiple comorbidities took a higher number of different medications and were on a more complex regimen, which was often correlated with a higher level of non-adherence [37]. On the other hand, patients with comorbidities were found to have a higher tendency to adhere to their medication, in a sense of illness perception [37]. However, without standard methodologies and terminologies it is quite challenging to compare studies providing numbers and percentages of different adherence parameters [36].

One important strength of this study is that the POAG diagnoses are based on evaluations of ophthalmologists, which ensures high data validity. The study uses a longitudinal cohort design with a random sample drawn from an officially processed generated register. The advantage of using such a register is the large number of patients included in study. This reduces possible bias due to self-selection and dropouts, which can be an issue in studies with voluntary participation of patients. An additional advantage is that populations in both private households and institutions are included.

However, there are also some limitations to such a study. We lack information about individual characteristics of people, such as their socio-economic status or ethnicity, but also about their basic health status prior to 2010. People who have undetected glaucoma diseases or who received a POAG diagnosis before 2010 and had no later diagnoses (false-negative diagnoses) are not included in our study population. False-positive diagnoses could not be completely excluded, however, we dealt with this by implementing a validation procedure which required recurrent diagnoses. Also, we used a restricted definition of adherence which exploits the frequency of collections of AGM from pharmacies over one-year periods. We also tested a different definition of adherence which only marginally affected the level of adherence and the results–as measured by the odds ratios of the panel regression—from the multivariate model. Thus, our results of the regression can be defined as robust. Nevertheless, as this is a model based on the numbers of prescriptions filled, no conclusion about the frequency of drop usage can be drawn.

The results of this study have demonstrated that a full one third of patients covered by the largest German health care insurer who have a confirmed diagnosis of open angle glaucoma are undertreated, especially in terms of lowering IOP using eye drops.

One has to assume that especially in this group of patients, who not even go to the pharmacy to fill their prescriptions, many are also unreliable about taking their medication regularly at all. These results are alarming, as for chronic diseases such as glaucoma, routine and regular medical care is absolutely essential to prevent disease progression [34].

### Health care implications

Each stakeholder in the system, the patient, the ophthalmologist, the insurance company, and the social system, is responsible for providing extra support to severely visually impaired patients. They all have an intrinsic interest in keeping this impairment rate as low as possible, and everyone involved is obliged to improve the standard of care. Ideally there should be close interaction of all interested parties but, according to our results, there is some doubt that this is the case. The complexity of the system, the lack of defined checkpoints, and probably the general feeling that patients are able to deal with their diagnosis may have created a false sense of security which must be addressed systematically. Knowing that global health care systems are very different and are nearly non-existent in some areas of the world, the following considerations might be helpful. In Germany, for instance, preventive medical eye care and especially patient education is inadequately reimbursed, which may play a role in our context.

The primary interaction between the ophthalmologist and the patient is one in which shared decision-making should create a reliable stimulus for the patient to buy and to use the medication as prescribed, e.g. by training the patient on how to apply the eye drops properly and by improving the transfer of knowledge about the severity of the disease. This interaction can be put in the context of personalized medicine, as each non-adherent patient may create his own understanding, such as suppression, economization, and even intellectualization of the disease. To some extent these factors can be identified by the doctor and addressed in the course of lifelong medical attendance and caretaking.

However, based on these results, the quality of interaction between doctors and patients is doubtlessly in need of improvement, and we presume many of the parties involved are not even aware of these deficiencies. A quotation of Stanislas Lem, the philosopher and writer, illustrates this phenomenon:

"*A persistent companion of perception is the ignorance of one's own ignorance*"

[38]

As long as the rules of privacy protection are met by health care providers, any kind of information that can be passed on to the doctor or the patient may help to improve the situation. Nevertheless, we should also define our position towards the patients’ own responsibility for their future lives, in which visual orientation and communication is of utmost importance. All these aspects are covered within the field of health literacy, which is essential to promote and encourage patients and providers to deal with the disease.

Furthermore, as forgetfulness undoubtedly plays a major role in adherence for patients with chronic illnesses, it would be worthwhile to stimulate the development of minimal invasive procedures such as selective laser trabeculaoplasty (SLT), minimally invasive glaucoma surgery (MIGS), and long-acting drug delivery systems for patients with POAG.

## Supporting information

S1 Table Panel regression model with identified co-factors of non-adherence on a quarter-specific basis, AOK data. (TIF)
