# Correction: Pharmacoepidemiological assessment of adherence and influencing co-factors among primary open-angle glaucoma patients—An observational cohort study

**DOI:** 10.1371/journal.pone.0193621

**Published:** 2018-02-23

**Authors:** The PLOS ONE Staff

The Funding statement is incorrect. The publisher apologizes for this error. The correct statement is: This work was supported by the Federal Ministry of Education and Research, Germany (grant number 03ZZ0904A).
