# Supplementary figures and images for: Pharmacoepidemiological assessment of adherence and influencing co-factors among primary open-angle glaucoma patients—An observational cohort study
Source: PLoS One. 2018 Jan 12;13(1):e0191185. doi: 10.1371/journal.pone.0191185 (PMC5766149; doi:10.1371/journal.pone.0191185)

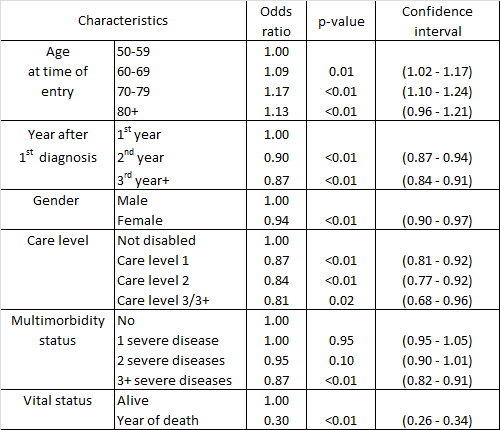

Supplement: S1 Table — (TIF) [file pone.0191185.s001.tif]
